# Supplementary material for: Restoration of high-sensitivity and adapting vision with a cone opsin
Source: Nat Commun. 2019 Mar 15;10:1221. doi: 10.1038/s41467-019-09124-x (PMC6420663; doi:10.1038/s41467-019-09124-x)
Supplement: Supplementary file 1 — Supplementary Information [file 41467_2019_9124_MOESM1_ESM.doc]

Restoration of high-sensitivity and adapting vision with a cone opsin

Berry et al.

**- Supplementary Material**

- **Comparing light sensitivity of optogenetic probes**
- **Electrophysiology and light stimulation for *in vivo* brain recordings**
- ***In vivo* brain recording data acquisition and analysis**
- **Animal Behavior Tasks**
- **Passive Avoidance - Open Field Test**
- **Visually Cued Fear-conditioning Paradigm**
- **Modified Active Avoidance Protocol**

**- Supplementary Figures and Legends**

**Supplementary Figure 1. MW-opsin expresses in *rd1* mouse retina**

**Supplementary Figure 2. Rhodopsin expresses in *rd1* mouse retina**

**Supplementary Figure 3. Transduction efficiency of MW-opsin in *rd1* retina**

**Supplementary Figure 4. Optogenetic threshold response compared to natural illumination**

**Supplementary Figure 5. Sensitivity and response rundown of Rhodopsin and MW-opsin**

**Supplementary Figure 6. Contrast detection in retina of MW-opsin expressing *rd1* mouse**

**Supplementary Figure 7. *In vivo* responses in V1 of *rd1* mouse expressing MW-opsin**

**Supplementary Figure 8. Contrast detection *in vivo* in MW-opsin expressing *rd1* mouse**

**Supplementary Figure S9. Temporal properties of *in vivo* V1 responses**

**Supplementary Figure 10. Light avoidance and learned pattern discrimination behaviors**

**Supplementary Figure 11. Location preference in discrimination task**

**Supplementary Figure 12. Light adaptation in excised retina and mouse visual behavior**

**Supplementary Table 1. Statistical significance of avoidance and learned behaviors**

**Supplementary Methods**

**Comparing light sensitivity of optogenetic probes**

The natural sensitivity of human vision covers a wide range, from scotopic (dim 10^4^ - 10^11^ photons cm^−2^ s^−1^) to photopic (bright 10^10^ – 10^17^ photons cm^−2^ s^−1^), allowing perception under dynamic lighting conditions. To relate the sensitivity of MW-opsin to that of other optogenetic systems and of normal vision, we measured natural light intensities under various outdoor and indoor conditions using a portable power meter (Thorlabs), compared them to our experimental paradigms and to light sensitivity thresholds (lowest measurable light responses) reported in the literature using monochromatic wavelengths of light at or near peak functional maxima in retinal explants isolated from *rd* mice to which the optogenetic probe was delivered via AAV transfection (Supplementary Figure 4).

**Electrophysiology and light stimulation for *in vivo* brain recordings**

*In vivo* recordings were performed as previously described by Veit et. al. (2017)^7^. Mice were anesthetized with isoflurane (2.5% vapor concentration). The scalp was removed, the fascia retracted, and the skull lightly etched with a 27 gauge needle. Following application of Vetbond to the skull surface, a custom stainless steel headplate was fixed to the skull with dental cement (Metabond). Mice were allowed to recover from surgery for at least 2 days. Then mice were habituated for 2–5 days to head-fixation on a free-spinning circular treadmill. On the day of recording, mice were briefly anesthetized with isoflurane (2%), the skull over V1 was thinned, and a small (<250 μm) craniotomy was opened over the primary visual cortex with a fine needle. A 16-channel linear electrode array with 25 micron spacing (NeuroNexus, A1x16-5mm-25-177-A16) was guided into the brain using a micromanipulator (Sensapex) and a stereomicroscope (Leica). Electrical activity was amplified and digitized at 30 kHz (Spike Gadgets), and stored on a computer hard drive. The cortical depth of each electrical contact was determined by zeroing the bottom contact to the surface of the brain. The electrode was inserted close to perpendicular to the brain surface.

Visual stimuli were generated with Psychophysics Toolbox ^8^ running on an Apple Mac Mini and were presented on a gamma corrected 23-inch Eizo FORIS FS2333 LCD display with a 60-Hz refresh rate. The monitor was centered 15cm from the right eye of the mouse covering roughly 108 by 61 degrees of visual angle. Mice were dark adapted for 7-10 minutes before every block of recordings. Two different stimulation paradigms were used: 1) Contrast: Mice were presented with 500ms full screen flashes at 0.5Hz of four different luminance levels (15, 25, 50 and 100% of maximal luminance) that were repeated 10 times in different random orders. 2) Flicker: Mice were presented with 20 500ms full screen flashes (maximum luminance 116 µW cm^-2^) at 0.0167 Hz (one per minute) or 100 flashes at 0.5, 1, 2 and 4 Hz in separate blocks.

***In vivo* cortical recording data, acquisition and analysis**

Spiking activity was extracted by filtering the raw signal between 800 and 7000 Hz. Spike detection was performed using the UltraMega Sort package^9^. Detected spike waveforms were sorted using the MClust package (http://redishlab.neuroscience.umn.edu/MClust/MClust.html). Waveforms were first clustered automatically using KlustaKwik and then manually corrected to meet criteria for further analysis. Units with more than 2% of their individual waveforms violating a refractory period of 2ms were classified as multi-units. The depth of each unit was assigned based on the calculated depth of the electrode on the array that exhibited its largest amplitude sorted waveform. Firing rates were computed by counting spikes in a two second window starting 250 ms after the onset of the visual stimulus for Fig. S9b and in a 5s window starting directly after stimulus onset for Fig. S10c. Traces for Peri-stimulus time histograms (PSTH)s were generated by binning the spiking response into either 20ms (Fig. S8) 50ms (S9) or 25ms (Fig. S10) bins and smoothing the resulting traces with a moving average filter. For Fig, S10, modulation amplitude was calculated on trial averaged spiking responses binned at 25ms in a 2, 1, 0.5 or 0.25s window for 0.5, 1, 2 and 4Hz flicker respectively. Corresponding baseline modulation amplitudes were generated from trial averaging the same number of same-length windows from before the onset of the first visual stimulus (during the dark adaptation period). Local field potentials were extracted by low pass filtering the raw signal, sampled at 30 kHz, below 200 Hz and subsequent down-sampling to 1 kHz.

**Animal Behavior Tasks**

Prior to treatment, animal cohorts were divided randomly to age-matched control groups and treated groups. Animals were run in small batches, with groups of control and test animals run in alternation. Groups within a cohort were run in different weeks to ensure that environmental changes within the animal facility testing room did not affect outcomes. Additionally, in some experimental rounds the control group was run first followed by the test group and in others the reverse sequence was used. All experiments were done using computer data analysis. Behavior was measured by IR sensors or video by automated routines that track animal position. The experimenter was not blind to the identity of the animals.

# Passive Avoidance - Open Field Test

The open field test was performed as described previously ^6, 10^ but with a difference in the source of illumination. A two-compartment (light and dark) shuttle box (Coulbourn Instruments) allows the mouse to move freely through a small opening that connects the two compartments. The light compartment was illuminated by a LED panel over the compartment. White light as well as wavelengths of 535nm and 470nm were used at light intensity of ~100 μW cm^-2^ (white light) and 25-0.5 μW cm^-2^ (blue and green light) was homogeneously distributed throughout the floor. Day 1 - mice were transferred into the testing box, and allowed to habituate to the new environment for up to 45 min (without illumination) until reaching equal exploration of each side. Mice were then retuned in to their home cage and then tested individually. Day 2 - Mice were placed in the light compartment and were given a maximum of 3 min to discover that there is a second compartment. A 15-min trial began when they crossed into the dark compartment, and time spent in the light was recorded. Mice that crossed the opening only once and stayed in the dark compartment for entire time were disqualified. For adaptation experiments mice were pre-exposed to 1,4, or 8 hrs of white light (white light; 1 mW cm^-2^ / 535nm spectral component; 50 μW cm^-2^) or dark exposure conditions then immediately performed the open field test. Animals movements were tracked using IR sensors on the shuttle box. Time spent on either side was collected and analyzed using the Graphic State, and Graphic State RT programs (Coulbourn Instruments).

**Visually Cued Fear-conditioning Paradigm**

Fear conditioning experiments were performed using Coulbourn shock chambers- Coulbourn Habitest chamber with test cage (Coulbourn Instruments, PA) with an LED screen mounted to the ceiling. On day 1 - animals were brought into the testing room in their home cages and then individually acclimated to clean Coulbourn shock chambers for 30 minutes. On day 2-3 animals were subjected to paired or unpaired light cued fear conditioning, consisting of 5 minutes habituation to the chamber (dark) followed by three shock trials at 0.7 mA over a span of 15 min. For paired trials, the transition from either static or flashing (2Hz) coincided with a brief foot shock at a 2 seconds intershock-interval (for 10 sec). For unpaired trials, animals also received shocks but were independent of the stimulus transition. These brief, low current shocks provided the minimal aversive stimuli to create a fearful memory associated with patterned light. On day 4 the flooring of box was replaced with plastic instead of shock grating. Mice were habituated to the chamber for 5 minutes and subjected to the same light stimulation protocol as on day 2 and 3 but without shock. Freezing behavior in anticipation of the shock was recorded by Coulbourn’s FreezeFrame software and normalized to movement behavior gathered before the stimulation. The performance was then compared between paired and unpaired cohorts in order to determine if a fear response was conditioned to the stimulus transition.

## Modified Active Avoidance Protocol

Avoidance protocols were done using Coulbourn shuttle box (H10-11M-SC) Coulbourn Habitest Isolation Cubicle (H10-24), and the Graphic State and Graphic State RT software (Coulbourn Instruments, PA). On Day 1 - animals were acclimated to darkened procedure room for 30 minutes in their home cages, followed by individual acclimation to darkened shuttle box until animals were non-fearful and spending approximately equal amounts of time on each side of shuttle box (exploratory behavior). On day 2-3 tablet screens were mounted in shuttle cage wall and displayed two distinct images of equal shape, size, light intensity. Each run began when the animal moved to the other side of shuttle box from where it was placed. Each run was 15 minutes long. The aversive image side was paired with a foot shock of 0.7mA at intervals of 5 seconds until the animal returned to the “safe” side. Any animal remaining on the aversive side for more than 60 seconds was removed from cage and that run was discarded. On Day 4 the light patterns was reversed (so the aversive screen is on what was formerly the “safe” side) to avoid a bias for location rather than pattern. Additionally, the flooring of the cage is replaced with plastic instead of shock grating. This ensures that the only related association with training day is the light stimuli. Again, animals were run for 15 minutes and time spent on each side was recorded. For light adaptation experiments, the same protocol was followed except that on day 4, the recall phase was preceded with either a light (65W white bulb) or dark adaptation phase for one hour. Tablet screens were dimmed or brightened to test adaptation to the different light intensities. Visual discrimination optical angle calculations were performed using the parameters of the behavioral shuttle cage (15.24x17.76 cm), the distance from decision point (divider), the mounted LCD panel (18.85 cm), and the parameters of the stimulus pattern (6 – 1 cm between parallel lines) using the optical (physical) angle equation. The comparison of acuity in *rd1* mouse expressing MW-opsin in RGCs and published values on sighted, *wt* animals was based on the following values, calculation and references. Distance from stimulus to decision point (L) = 18 cm. Distance between the lines (D) = 6 cm. Visual angle (V) = 2tan^-1^((D/2)/(L)) = 18 degrees = 0.33 radians. Cycles per degree = 1/V ~ 0.056 cpd

**Supplemental Figures and Legends**

**
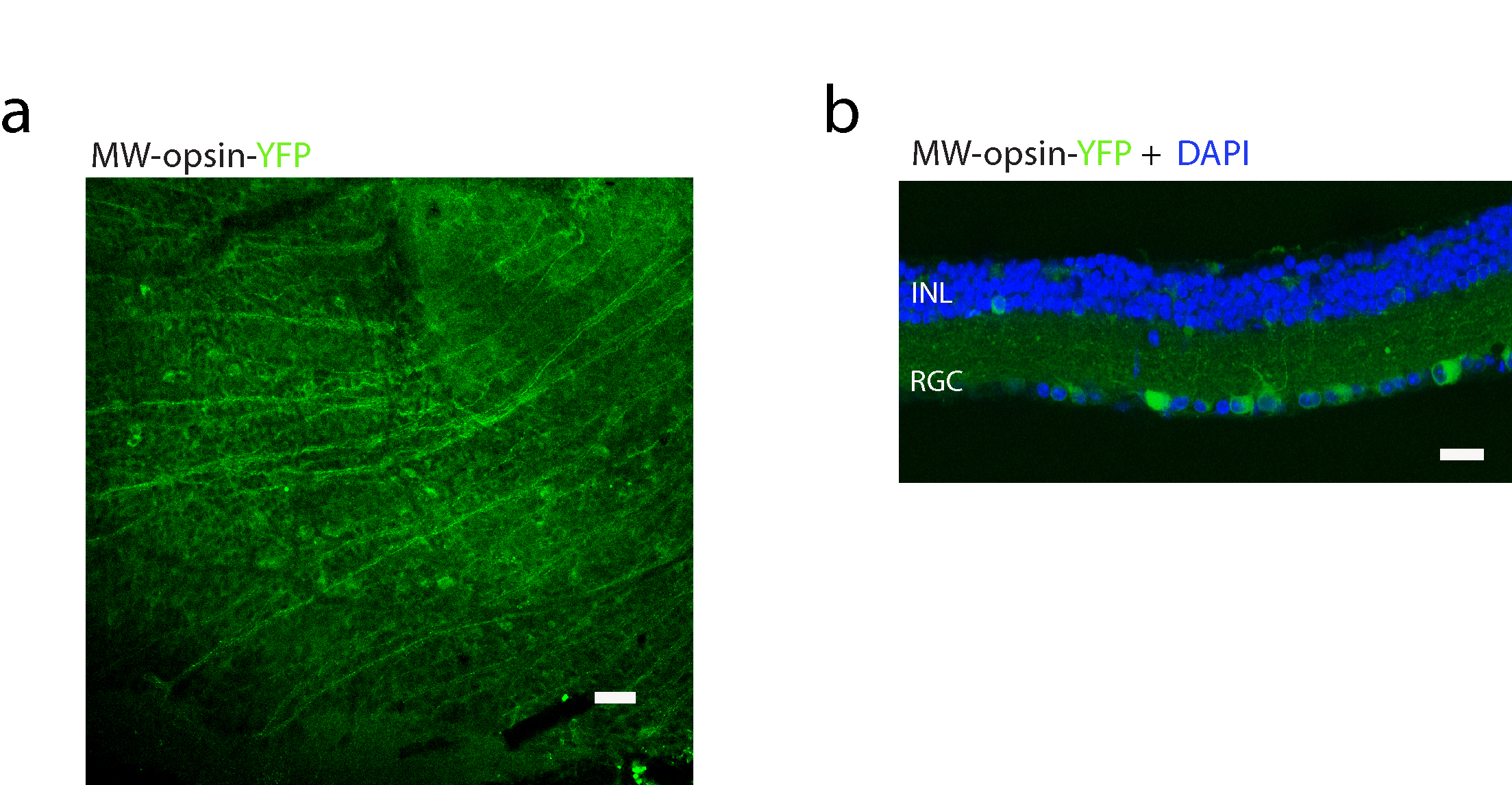
**

**Supplementary Figure 1. MW-opsin expresses in *rd1* mouse retina**

Flat mount (a) and slice (b) confocal images of MW-opsin-YFP in RGCs of *rd1* mouse retina 4-6 wks after intravitreal injection of *AAV2/2-hSyn-MW-opsin-YFP*. YFP fluorescence (green) and DAPI stain of nuclei (blue). INL=Inner nuclear layer, RGC=Retinal ganglion cell layer. Scale 40 μm (a) and 20 μm (b).


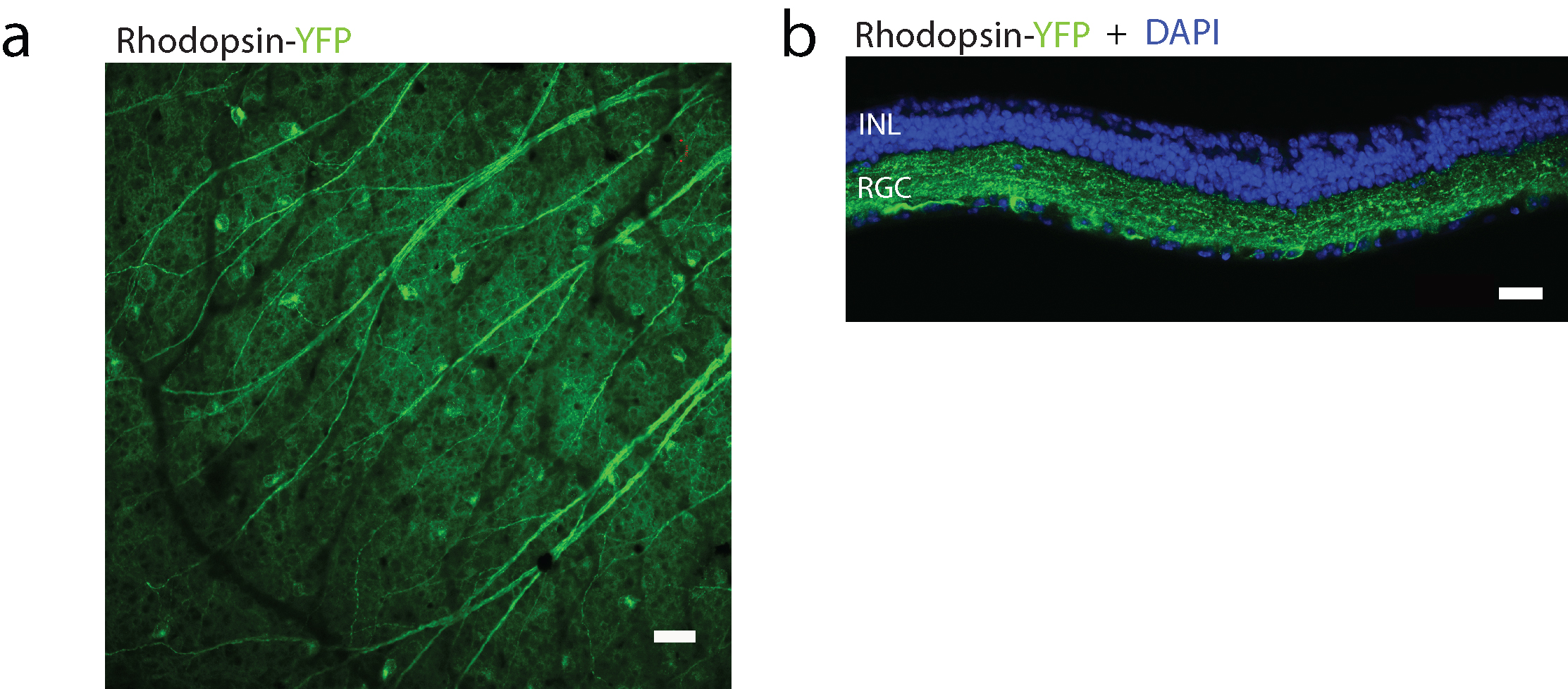


**Supplementary Figure 2. Rhodopsin expresses in *rd1* mouse retina**

Flat mount (a) and slice (b) confocal images of rhodopsin-YFP in RGCs of *rd1* mouse retina 4-6 wks after intravitreal injection of *AAV2/2-hSyn-rhodopsin-YFP*. YFP fluorescence (green) and DAPI stain of nuclei (blue). INL=Inner nuclear layer, RGC=Retinal ganglion cell layer. Scale 40 μm (a) and 20 μm (b).

**
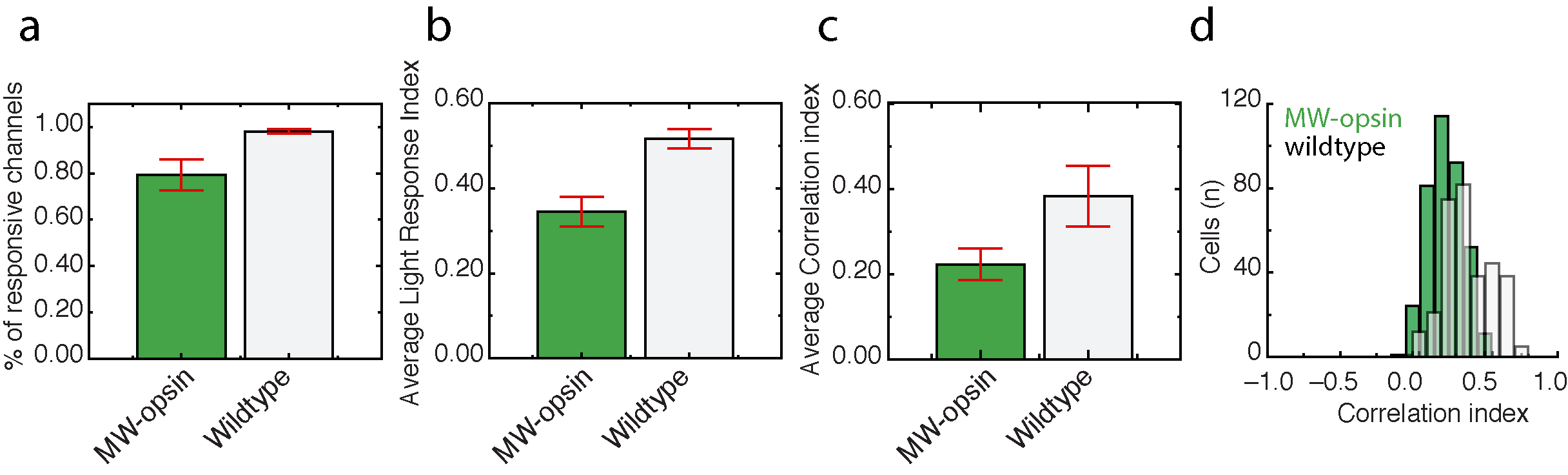
**

**Supplementary Figure 3. Transduction efficiency of MW-opsin in *rd1* retina**

(a) Average percent of channels identified in retina of *rd1* mouse expressing MW-opsin that display light responsiveness in MEA recordings. Light responsiveness defined as Light Responsive Index (LRI) >0.1, parameters established in Tochitsky et al. (2014)^5^, Gaub. et al (2014)^6^ and Berry et al. (2017)^11^. (LRI = peak firing rate in light - average firing rate in dark / peak firing rate in light + average firing rate in dark). (b) Average normalized response across retinas). (a,b) N=8 *rd1* retinas, N=5 *wt* retinas. (c) Average cross-correlation values in MW-opsin *rd1* (n=370 cells, N=3 retinas) and in wildtype (*n* = 237 cells, N = 3 retinas). Wavelength: λ = 535 nm filter, 2x10^-1^ mW cm^-2^ Values are mean + SEM. Cross-correlation of all light-sensitive units in a period 1 s before to 2 s after the light pulse. (d) Correlative value distributions for light sensitive units within the same retina for MW-opsin (n=370 cells, N=3 retinas) and *wt* retina (n=237 cells, N=3 retinas).


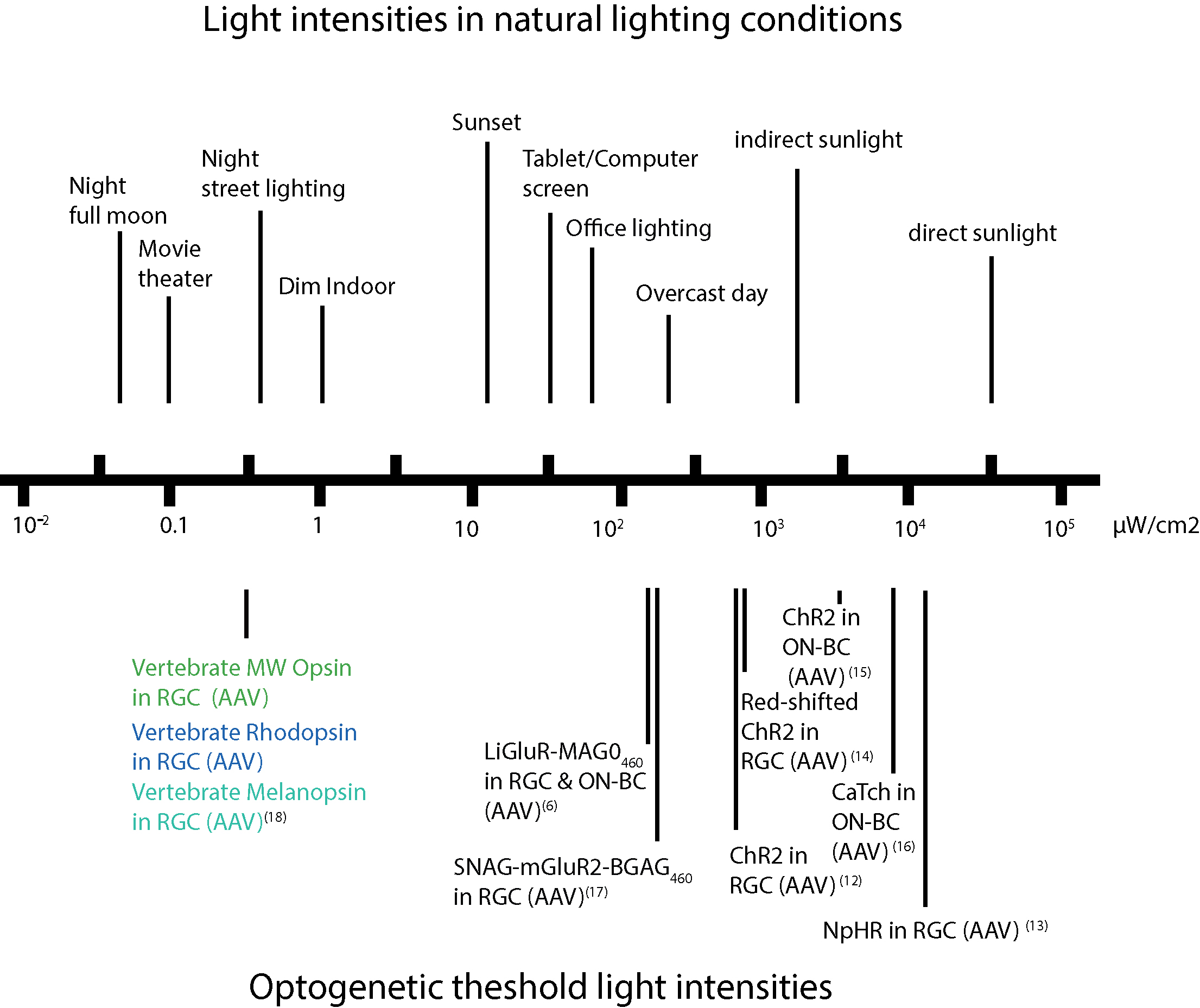


**Supplementary Figure 4. Optogenetic threshold response compared to natural illumination**

Comparison of threshold sensitivity of natural vision (top) to that of various optogenetic systems introduced to a defined set of surviving neurons in a retina following degeneration of photoreceptor cells (bottom). Mammalian MW-opsin (green), rhodopsin (blue) and melanopsin (cyan) are ~1000x more sensitive than microbial Channelrhodopsin2 and Halorodopsin. Threshold for alternative optogenetic approaches from Bi et al. (2006)^12^, Zhang et al (2009)^13^, Sengupta et al. (2016)^14^, Dodoucci et al. (2011)^15^, Gaub et al. (2014)^6^, Cronin et al (2014)^16^, Berry et al. (2017)^17^ De Silva et al. (2017)^18^.

(12) Bi et al. (2006) Channelrhodopsin expressed with AAV2-CAG promoter stimulated at 460nm in *rd1* mouse with sensitivity limit in RGCs = 2.2 × 10^15^ photons cm^−2^ s^−1^ ~ 1 mW cm^-2^.

(13) Zhang et al (2009) Halorhodopsin expressed with AAV2-CMV promoter stimulated at 555 – 575 nm band pass in *rd1* mouse with a sensitivity limit in RGCs = 5.8 × 10^16^ photons cm^−2^ s^−1^ ~ 20 mW cm^-2^.

(14) Sengupta et al. (2016) Red‐shifted channelrhodopsin expressed with AAV2-hSyn promoter stimulated at 595 nm in *rd1* mouse with a sensitivity limit in RGCs = 2.5 × 10^15^ photons cm^−2^ s^−1^ ~1 mW cm^-2^.

(15) Dodoucci et al. (2011) Channelrhodopsin expressed with AAV8-Y733F & SV-40 promoter stimulated at 450–490 nm band pass in *rd10* mouse with a sensitivity limit in ON-BCs = 4 × 10^16^ photons cm^−2^ s^−1^ ~ 17 mW cm^-2^.

(6) Gaub et al. (2014) LiGluR-MAG_460_ expressed with AAV2-hSyn and AAV2-4xGrm6 promoter stimulated at 445/20 nm in *rd1* mouse with a sensitivity limit in RGCs & ON-BCs = 7.1 × 10^14^ photons cm^−2^ s^−1^ ~ 0.3 mW cm^-2^.

(16) Cronin et al (2014) Channelrhodopsin expressed with AAV2/8BP2 & 4xGRM6 promoter stimulated at (unknow) in *rd1* mouse with a sensitivity limit in ON-BCs = 1 × 10^16^ photons cm^−2^ s^−1^ ~ 5 mW cm^-2^.

(17) Berry et al. (2017) SNAG-MGluR2 expressed with AAV2-hSyn promoter stimulated at 445/50 nm in *rd1* mouse with a sensitivity limit in RGCs = 1 × 10^14^ photons cm^−2^ s^−1^ ~ 0.5 mW cm^-2^.

(18) De Silva et al. (2017) expressed with AAV2/8(Y733F) & CMV enhancer/CBA promoter stimulated at 480/20 nm in *rd1* mouse with a Melanopsin sensitivity limit in RGCs = 1.20 × 10^12^ photons cm^−2^ s^−1^ ~ 0.5 µW cm^-2^.

**
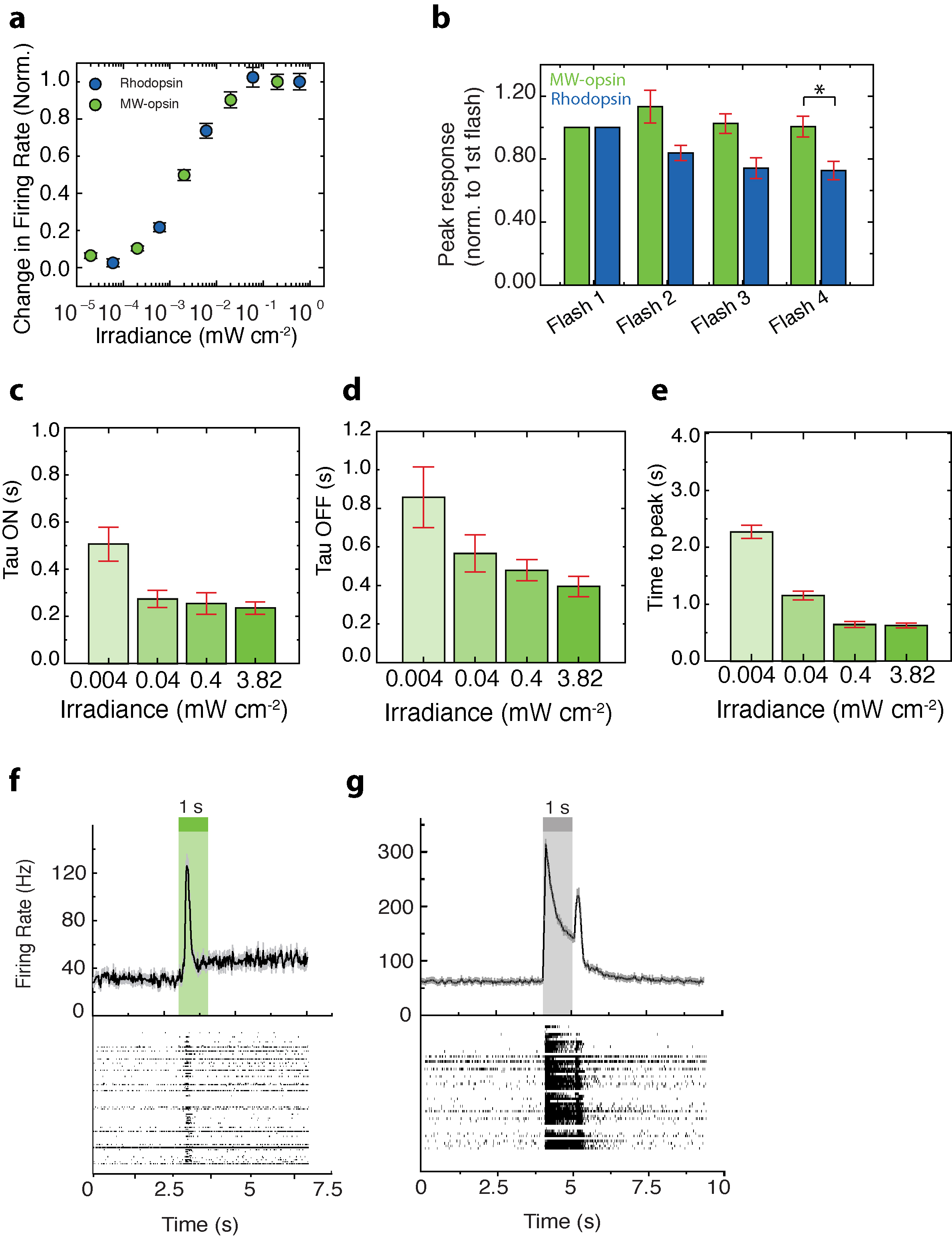
 Supplementary Figure 5. Sensitivity and response rundown of rhodopsin and MW-opsin**

**(a)** Light sensitivity of MW-opsin (N = 6 retinas) and rhodopsin (N = 4 retinas) in RGCs of *rd1* mouse retina. Peak firing rate normalized to maximum response.

**(b)** Peak response for 1^st^ and 4th light flash normalized to the first flash for MW-opsin (N = 4 retina) and rhodopsin (N = 8 retina) expressing retina. (student’s two-tailed t-tests, p<0.05)

**(c,e)** Dependence of light intensity (in seconds) of Tau ON (a), Tau OFF (b), and Time to peak (T-peak) (c) in *rd1* retina expressing MW-opsin n =111 cells, N=3 retina. All cells refer to sorted units. Values are mean, error bars are SEM. Wavelength: λ = 535 nm.

**(f,g)** Average response of RGC population with SEM in gray (top) and averaged raster plot over 5 light flashes of 1 sec duration (bottom) for *rd1* expressing MW-opsin (d; λ=510nm; n= 117 cells) and *wt* (e, white light; n= 50 cells).


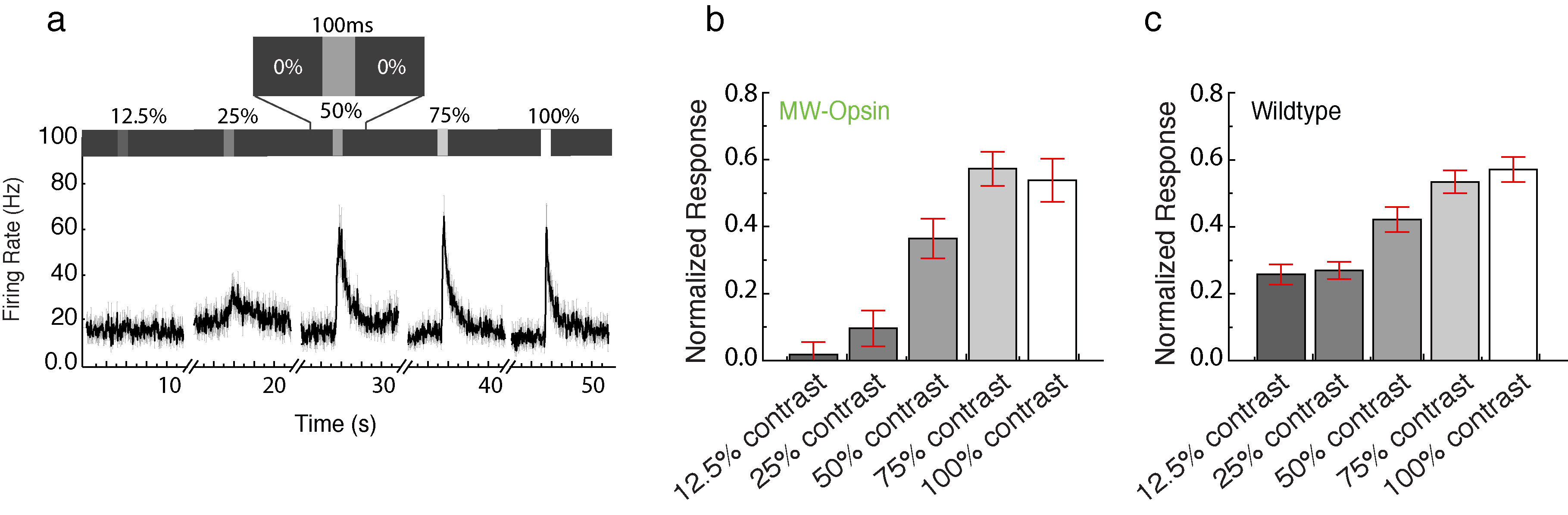


**Supplementary Figure 6. Contrast detection in retina of MW-opsin expressing *rd1* mouse**

MEA recording of responses to steps from dark to full-field gray scale of different intensities in example isolated retina from MW-opsin expressing *rd1* mouse (average of 14 channels) (a). (b,c) Normalized change in RGC firing rate in response to changes in contrast for *rd1* mice expressing MW-opsin (N = 2 retinas) (b) and *wt* mice (N = 2 retinas) (c) with error bars in SEM. 100% light= 25 µW cm^-2^.


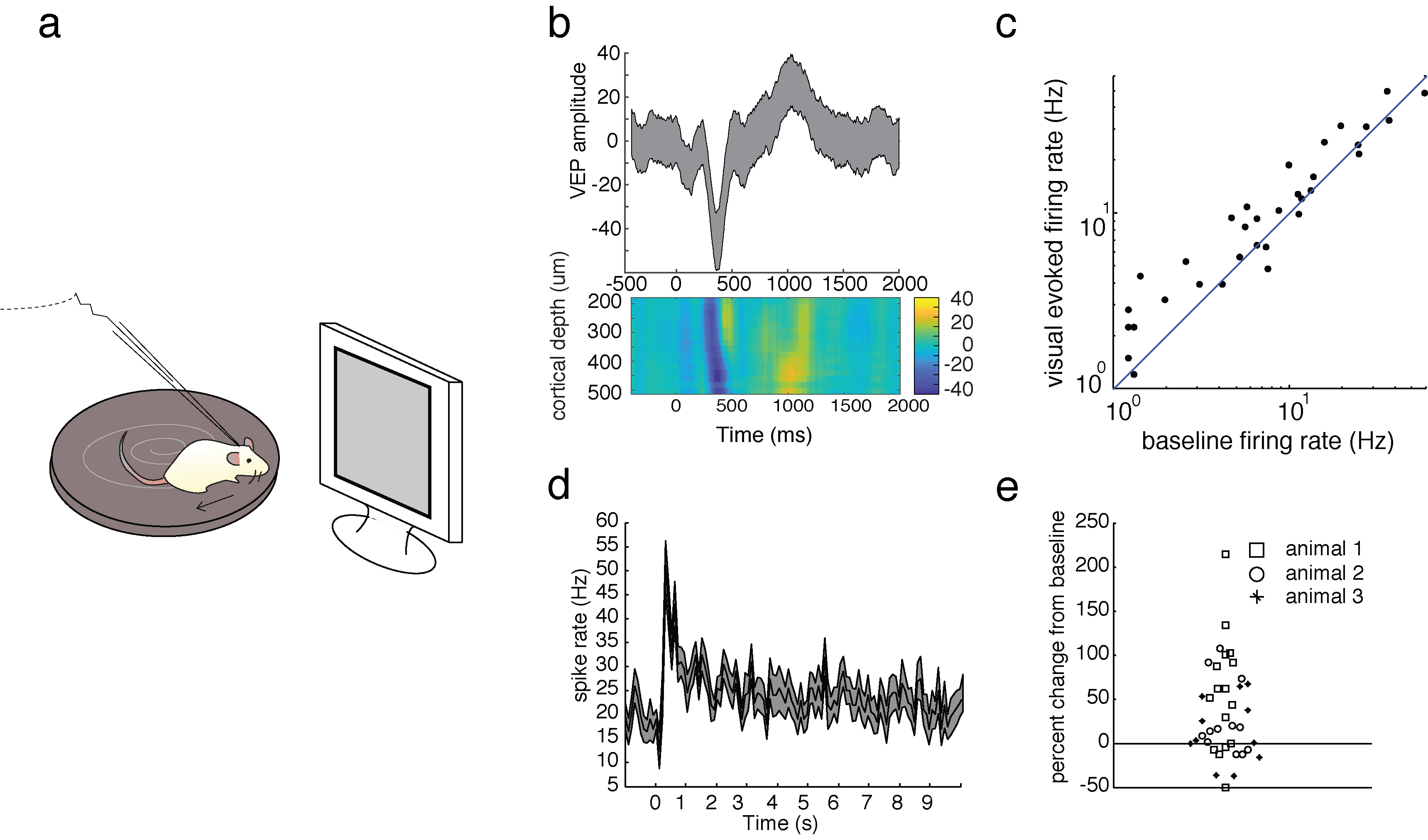


**Supplementary Figure 7. *In vivo* responses in V1 of *rd1* mouse expressing MW-opsin**

(a) Schematic of head-fixed mouse on a running wheel. Stimuli displayed on a standard computer monitor positioned within the visual field of one eye. (b) Responses to 500 ms light pulse. Top, representative visually evoked potential from layer 4 of V1 (average of 20 responses, shaded area represents mean ± SEM). Bottom, heat map of responses of individual units from all 16 electrodes of the linear electrode array across the depth of the visual cortex (average of 20 responses). (c) Scatter plot of light-evoked versus baseline firing rate for 39 units across 3 *rd1* mice expressing MW-opsin. (d) PSTH (binned at 50ms) of a representative unit in response to a light flash. (e) Plot of percent change of neuronal firing following stimulation in 39 units across 3 mice.


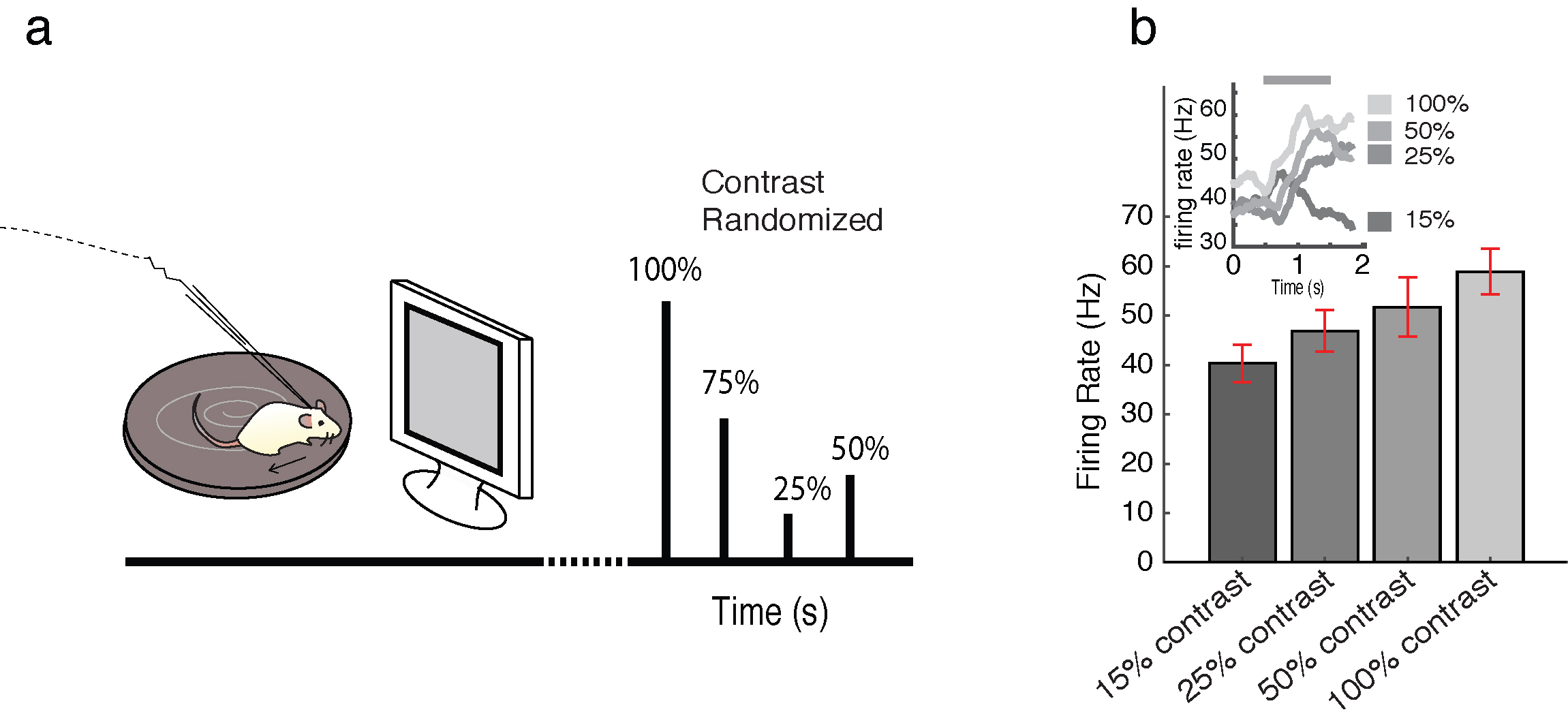


**Supplementary Figure 8. Contrast detection *in vivo* in MW-opsin expressing *rd1* mouse**

(a) Illustration of visual cortex recordings in awake free running *rd1* mouse expressing MW-opsin. Randomized contrast changes presented on a computer monitor placed within the animals’ visual field. (b) Average firing rate in response to full field contrast steps (500ms duration) recorded in layer 4 of V1. Inset shows PSTHs binned at 20ms and error in SEM. 100% light= 115 µW cm^-2^.


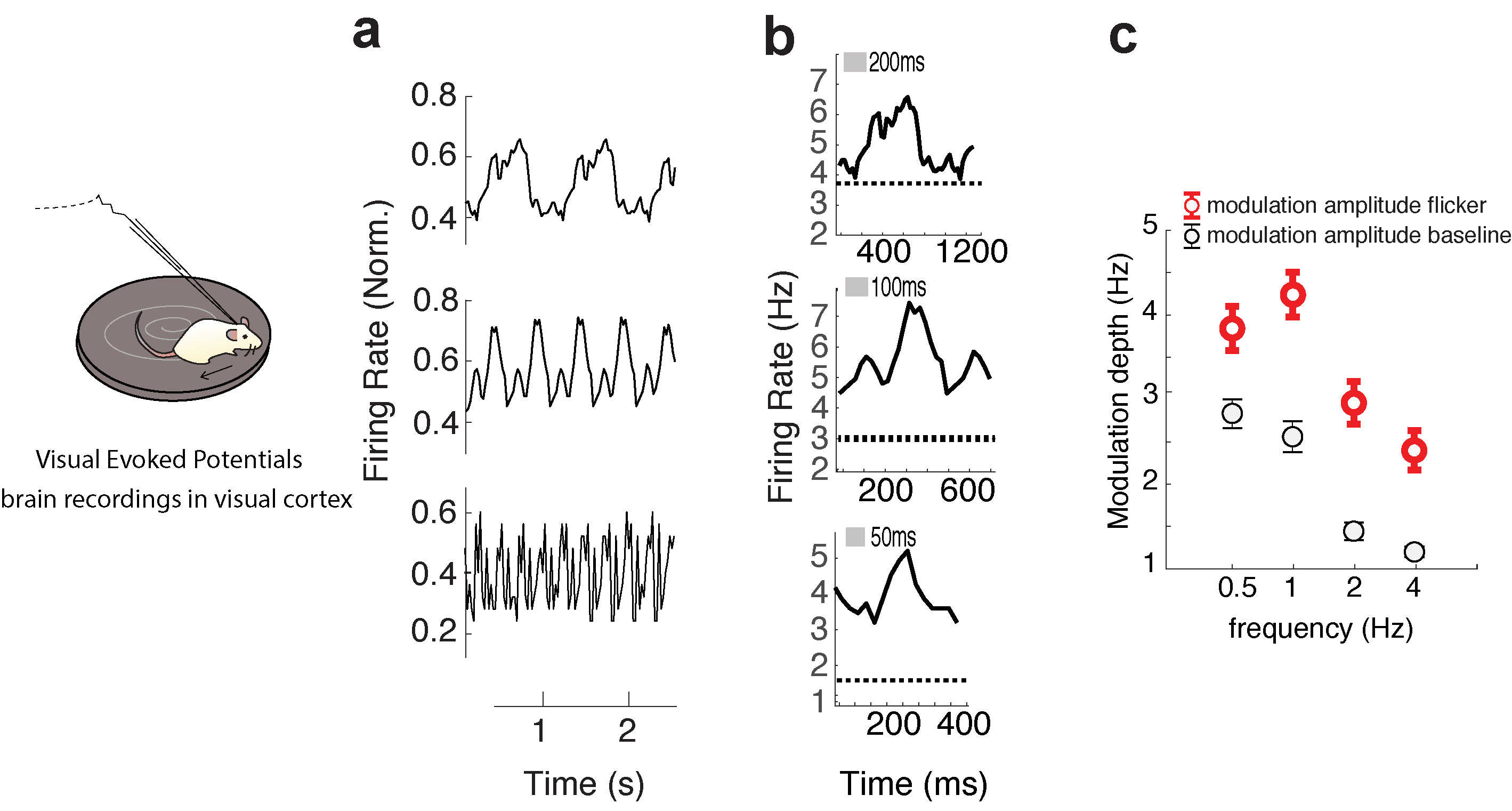


**Supplementary Figure 9. Temporal properties of *in vivo* V1 responses**

**(a-c)** Changes in neuronal firing in the visual cortex induced by full field flickering stimuli of 1, 2 and 4 Hz across 100 cycles and error bars in SEM.


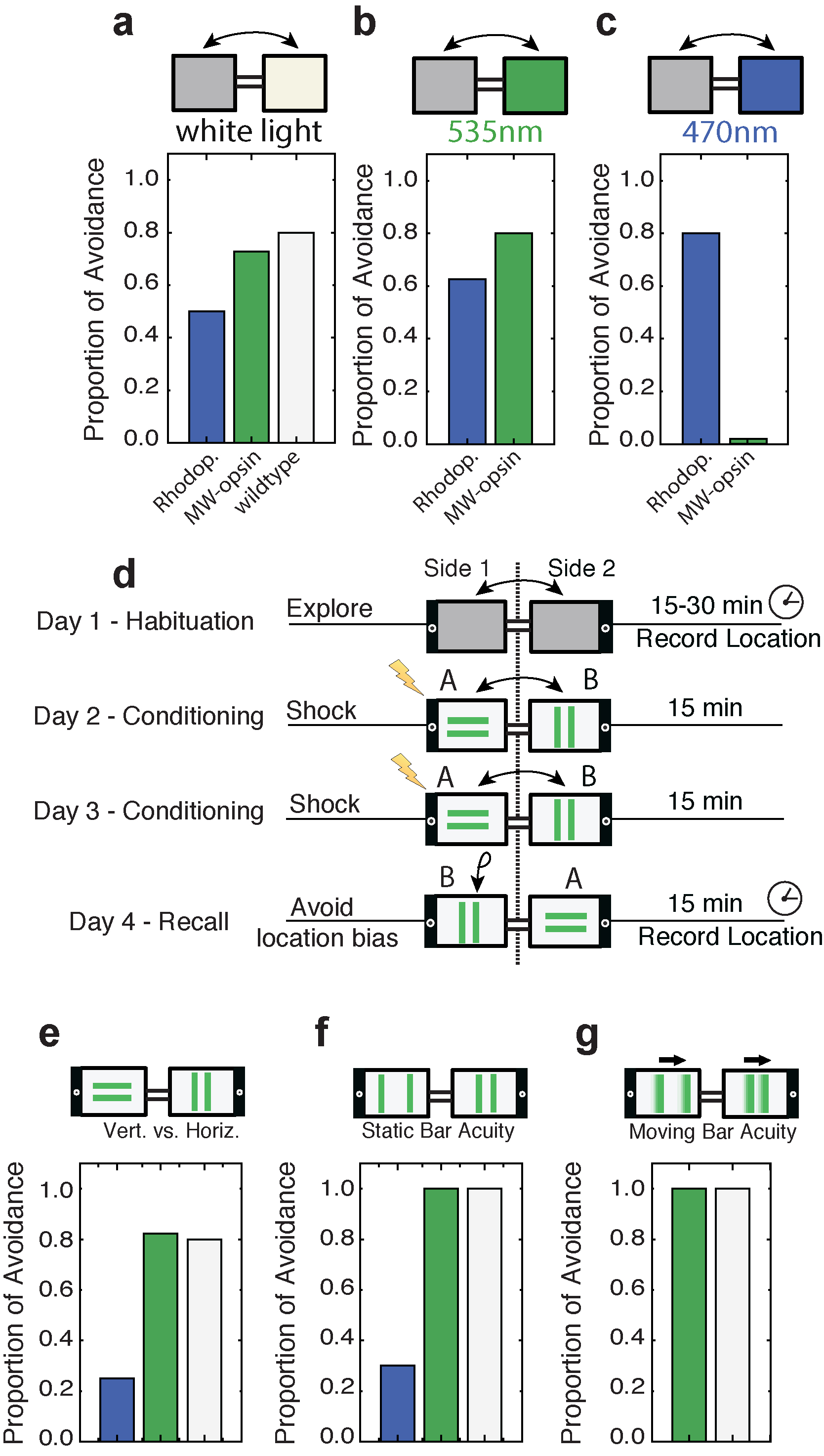


**Supplementary Figure 10. Light avoidance and learned pattern discrimination behaviors**

**(a-c)** Proportion of successful avoidance of the light compartment (proportion of successful avoidance trials), respectively, for a, b, and c in *rd1* expressing rhodopsin in RGCs (blue; n = 6,8 and 5 mice) or MW-opsin (green; n = 11,10, and 4 mice), and *wt* mice (white; n = 5 mice) when illuminated with either (a) white light (100 μW cm^-2^), (b) 1 μW cm^-2^ green light (535 nm) or (c) 1 μW cm^-2^ blue light (470 nm). Statistical significance assessed using Two-Sided Pearson's Chi-Square Test and One-Sided Fisher's Exact Test when applicable (See Supplementary Table 1). Success defined as avoidance greater than mean + S.D. of *rd1* untreated control mice (See Supplementary Methods)

**(d)** Schematic of pattern discrimination experiment. Mice habituated at day 1, exposed to electric shock in association with specific pattern of light (stimulus A/B) paired randomly in either chamber on days 2 and 3 and tested (time spent in each chamber) on day 4, in absence of shock with light patterns reversed to avoid location bias.

**(e-g)** Proportion of successful discrimination of the pattern paired with shock (proportion of successful avoidance trials) compared to *rd1* untreated controls. (e) Horizontal vs. vertical parallel bars. Discrimination of parallel static (f) or moving (g) bars at distances of 1 vs. 6 cm. Respectively for e,f and g: *rd1* rhodopsin (blue; n = 8(e), 6(f) mice), *rd1* MW-opsin (n = 17(e), 11(f), 6(g) mice) and *wt* (n = 5(e) ,6(f) ,9(g) mice). (25 μW cm^-2^). Statistical significance assessed using Two-Sided Pearson's Chi-Square Test and One-Sided Fisher's Exact Test when applicable (Supplementary Table 1). Success defined as avoidance greater than mean + S.D. of *rd1* untreated control mice (See Supplementary Materials & Methods).

**
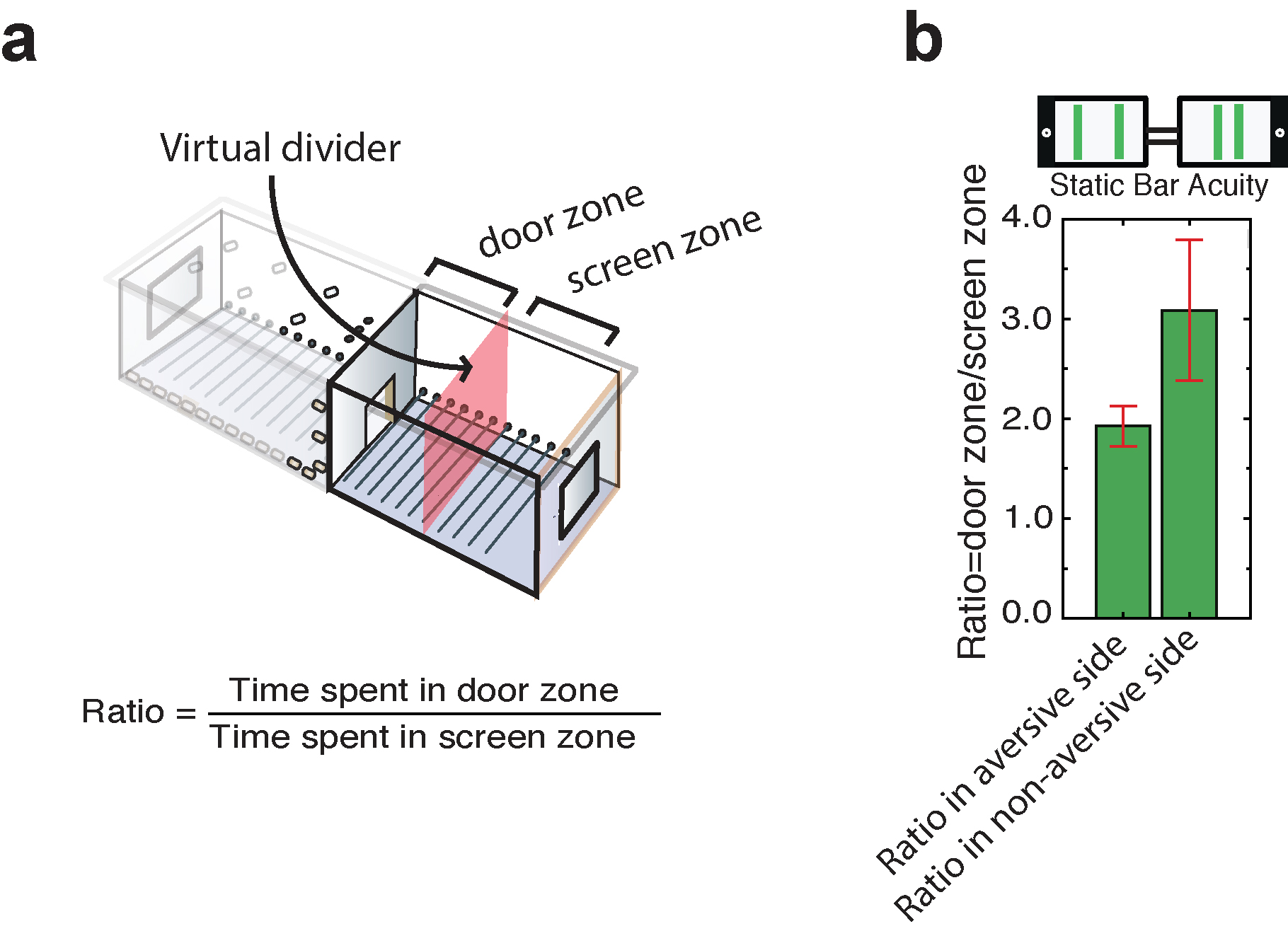
**

**Supplementary Figure 11. Location preference in discrimination task**

**(a)** Illustration of arena used in discrimination tasks, showing a virtual divider separating each compartment in half in order to determine the proportion of time spent near the the central divider between the two chambers versus near the screen (n=4 mice).

**(b)** Ratio of time spent in the zone close to central divider versus zone closest to the screen for the aversive and non-aversive sides reveals preference for zone closest to the central divider. Values are mean; error bars are SEM.


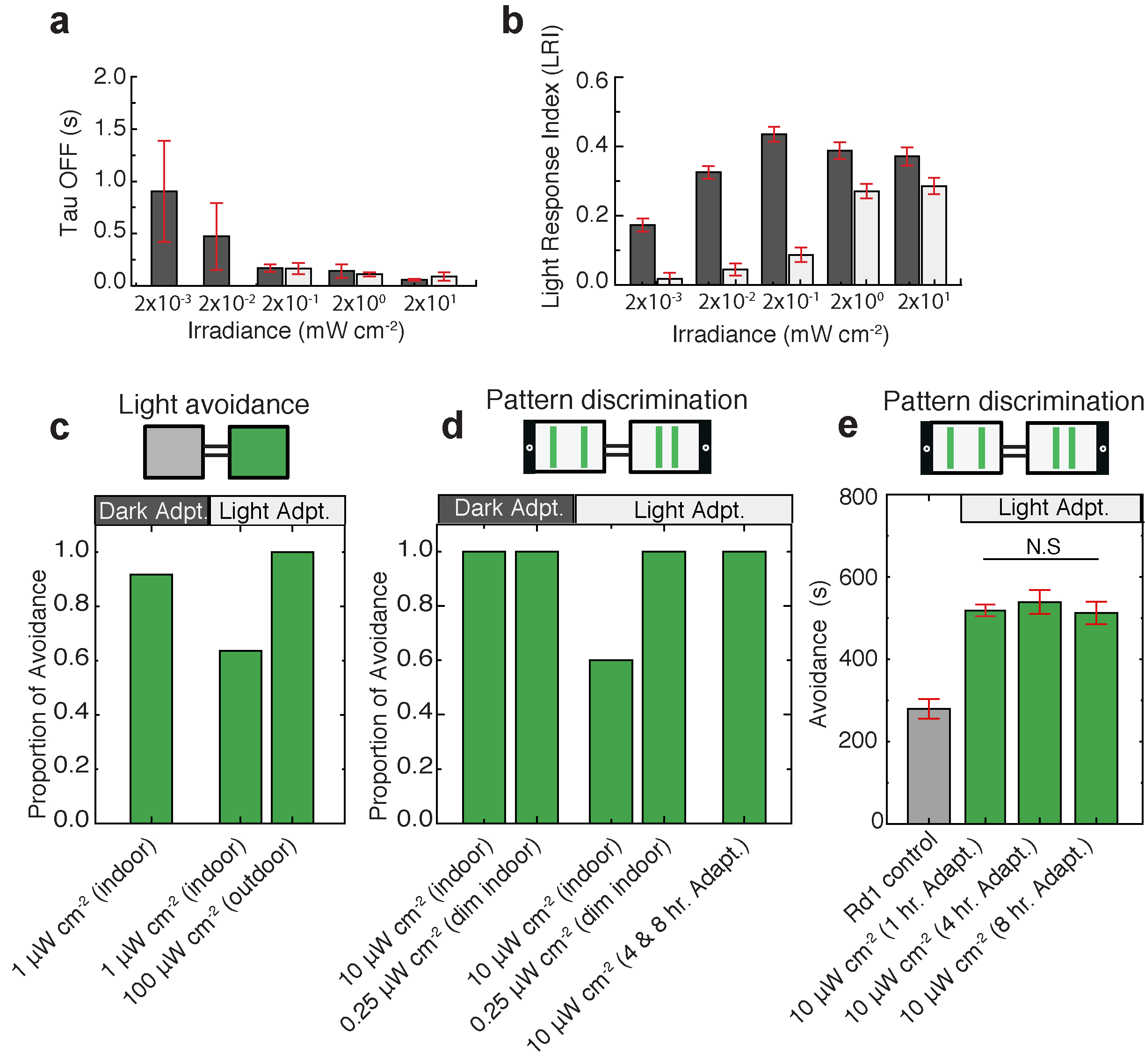


**Supplementary Figure 12. Light adaptation in excised retina and mouse visual behavior**

**(a-b)** MEA recordings showing light adaptation. Response in retina expressing MW-opsin to 100 ms light flashes ranging from 2 x 10^-3^ mW cm^-2^ to 2 x 10^1^ mW cm^-2^ following adaptation to dark or light.

(a) Light response decay (Tau OFF) as a function of flash intensity in dark *versus* light adapted condition (N = 3 retinas, n^c^ = 88 channels).

**(b)** Average (error bars are SEM) normalized Light response Index (LRI) at different flash intensities in same retina, first dark adapted and then light adapted (N = 3 retinas, n^c^ = 88 channels).

**(c)** Avoidance of the light compartment (proportion of successful avoidance trials) under indoor light (1 μW cm^-2^) following 1 hr. of adaptation to dark (n = 11 mice) compared with proportion of successful avoidance mice under indoor light (1 μW cm^-2^; n = 11 mice) and outdoor light (100 μW cm^-2^; n = 13 mice) following 1 hr. of adaptation to light (white light; 1 mW cm^-2^ / 535nm spectral component; 50 μW cm^-2^).

**(d)** Proportion of successful discrimination of the parallel bars spaced at distances of 1 *versus* 6 cm displayed at low (0.25 μW cm^-2^) or indoor (10 μW cm^-2^) light levels following 1 hr. of adaptation to dark (n = 11 mice at 0.25 μW cm^-2^, 8 mice at 10 μW cm^-2^) or light (n = 10 mice at 0.25 μW cm^-2^, 7 mice at 10 μW cm^-2^).

**(e)** Learned pattern discrimination of parallel bars spaced at distances of 1 *versus* 6 cm displayed at indoor (10 μW cm^-2^) light levels following 1, 4 or 8 hrs. of light adaptation (n=7,3,4) compared to unadapted *rd1* controls (error bars are SEM).

**Supplementary Table 1. Statistical significance of avoidance and learned behaviors** Success ratios were calculated for avoidance performance of condition behaviors (Figs. 3 and 4 and Supplementary Figs. 10 and 12e,f). To determine significance in differences between conditions a pairwise contingency table was then constructed, and a Two-Sided Pearson's Chi-Square Test was initially conducted. To correct for conditions with a small n, a One-Sided Fisher's Exact Test was also conducted. For some comparisons the Pearson's Chi-Square and Fischer's Exact Test were not appropriate comparisons as all results were successes in comparison to the rd1 control and both tests do not highlight if the comparisons are drawn from different probability distributions. These comparisons were denoted as N/A in Fisher’s Exact Test.

**Supplemental References**

1. Gaub BM, Berry MH, Holt AE, Isacoff EY, Flannery JG. Optogenetic Vision Restoration Using Rhodopsin for Enhanced Sensitivity. *Mol Ther* **23**, 1562-1571 (2015).

2. Broichhagen J*, et al.* Orthogonal Optical Control of a G Protein-Coupled Receptor with a SNAP-Tethered Photochromic Ligand. *ACS Cent Sci* **1**, 383-393 (2015).

3. Levitz J, Broichhagen, J., Leippe, P., Konrad, D., Trauner, D. and Isacoff, E.Y. Dual optical control and mechanistic insights into photoswitchable group II and III metabotropic glutamate receptors. *Proc Natl Acad Sci U S A*, (2017).

4. Roska B, Molnar A, Werblin FS. Parallel processing in retinal ganglion cells: how integration of space-time patterns of excitation and inhibition form the spiking output. *Journal of neurophysiology* **95**, 3810-3822 (2006).

5. Tochitsky I*, et al.* Restoring visual function to blind mice with a photoswitch that exploits electrophysiological remodeling of retinal ganglion cells. *Neuron* **81**, 800-813 (2014).

6. Gaub BM*, et al.* Restoration of visual function by expression of a light-gated mammalian ion channel in retinal ganglion cells or ON-bipolar cells. *Proc Natl Acad Sci U S A* **111**, E5574-5583 (2014).

7. Veit J, Hakim R, Jadi MP, Sejnowski TJ, Adesnik H. Cortical gamma band synchronization through somatostatin interneurons. *Nat Neurosci* **20**, 951-959 (2017).

8. Brainard DH. The psychophysics toolbox. *Spatial Vision* **10**, 433-436 (1997).

9. Hill DN, Mehta SB, Kleinfeld D. Quality Metrics to Accompany Spike Sorting of Extracellular Signals. *Journal of Neuroscience* **31**, 8699-8705 (2011).

10. Lin B, Koizumi A, Tanaka N, Panda S, Masland RH. Restoration of visual function in retinal degeneration mice by ectopic expression of melanopsin. *Proceedings of the National Academy of Sciences of the United States of America* **105**, 16009-16014 (2008).

11. Berry M*, et al.* Restoration of Patterned Vision with an Engineered Photo-Activatable G Protein-Coupled Receptor. *Nat Commun*, (2017).

12. Bi A*, et al.* Ectopic expression of a microbial-type rhodopsin restores visual responses in mice with photoreceptor degeneration. *Neuron* **50**, 23-33 (2006).

13. Zhang Y, Ivanova E, Bi A, Pan ZH. Ectopic expression of multiple microbial rhodopsins restores ON and OFF light responses in retinas with photoreceptor degeneration. *J Neurosci* **29**, 9186-9196 (2009).

14. Sengupta A*, et al.* Red‐shifted channelrhodopsin stimulation restores light responses in blind mice, macaque retina, and human retina. *EMBO Molecular Medicine* **8**, 1248-1264 (2016).

15. Doroudchi MM*, et al.* Virally delivered channelrhodopsin-2 safely and effectively restores visual function in multiple mouse models of blindness. *Mol Ther* **19**, 1220-1229 (2011).

16. Cronin T*, et al.* Efficient transduction and optogenetic stimulation of retinal bipolar cells by a synthetic adeno-associated virus capsid and promoter. *EMBO Molecular Medicine* **6**, 1175-1190 (2014).

17. Berry MH*, et al.* Restoration of patterned vision with an engineered photoactivatable G protein-coupled receptor. *Nat Commun* **8**, 1862 (2017).

18. De Silva SR*, et al.* Long-term restoration of visual function in end-stage retinal degeneration using subretinal human melanopsin gene therapy. *Proc Natl Acad Sci U S A*, (2017).
