# Supplementary material for: Restoration of high-sensitivity and adapting vision with a cone opsin
Source: Nat Commun. 2019 Mar 15;10:1221. doi: 10.1038/s41467-019-09124-x (PMC6420663; doi:10.1038/s41467-019-09124-x)
Supplement: Supplementary file 3 — Reporting Summary [file 41467_2019_9124_MOESM3_ESM.pdf]

nature research

Corresponding author(s): Ehud Isacoff

Last updated by author(s): Jan 4, 2019

# Reporting Summary

Nature Research wishes to improve the reproducibility of the work that we publish. This form provides structure for consistency and transparency in reporting. For further information on Nature Research policies, see Authors & Referees and the Editorial Policy Checklist.

## Statistics

For all statistical analyses, confirm that the following items are present in the figure legend, table legend, main text, or Methods section.

| n/a | Confirmed | |
|---|---|---|
| ☐ | ☒ | The exact sample size (*n*) for each experimental group/condition, given as a discrete number and unit of measurement |
| ☐ | ☒ | A statement on whether measurements were taken from distinct samples or whether the same sample was measured repeatedly |
| ☐ | ☒ | The statistical test(s) used AND whether they are one- or two-sided<br>*Only common tests should be described solely by name; describe more complex techniques in the Methods section.* |
| ☐ | ☒ | A description of all covariates tested |
| ☐ | ☒ | A description of any assumptions or corrections, such as tests of normality and adjustment for multiple comparisons |
| ☐ | ☒ | A full description of the statistical parameters including central tendency (e.g. means) or other basic estimates (e.g. regression coefficient) AND variation (e.g. standard deviation) or associated estimates of uncertainty (e.g. confidence intervals) |
| ☒ | ☐ | For null hypothesis testing, the test statistic (e.g. *F*, *t*, *r*) with confidence intervals, effect sizes, degrees of freedom and *P* value noted<br>*Give P values as exact values whenever suitable.* |
| ☒ | ☐ | For Bayesian analysis, information on the choice of priors and Markov chain Monte Carlo settings |
| ☒ | ☐ | For hierarchical and complex designs, identification of the appropriate level for tests and full reporting of outcomes |
| ☒ | ☐ | Estimates of effect sizes (e.g. Cohen's *d*, Pearson's *r*), indicating how they were calculated |

*Our web collection on statistics for biologists contains articles on many of the points above.*

## Software and code

Policy information about availability of computer code

| | |
|---|---|
| Data collection | Retinal activity on the MEA was sampled at 25 kHz filtered between 100 and 2,000 Hz and recorded using MC_rack software (Multi Channel Systems). |
| Data analysis | For MEA analysis: Voltage traces were converted to spike trains offline and the spikes recorded at each electrode were sorted into single units, which we defined as “cells,” via principal component analysis using Offline Sorter (Plexon-64bit) with each electrode commonly identifying 1-3 cells. Single-unit spike clusters or channels were exported to MATLAB (MathWorks) and were analyzed and graphed with custom software.<br><br>For open field exploration behavior analysis: Videos were analyzed for the latency to arrive at and explore each object, the velocity of travel (cm/s) and distance travelled (cm) using Noldus Technology Ethosvision XT v13.5. |

For manuscripts utilizing custom algorithms or software that are central to the research but not yet described in published literature, software must be made available to editors/reviewers. We strongly encourage code deposition in a community repository (e.g. GitHub). See the Nature Research guidelines for submitting code & software for further information.

## Data

Policy information about availability of data

All manuscripts must include a data availability statement. This statement should provide the following information, where applicable:

- Accession codes, unique identifiers, or web links for publicly available datasets
- A list of figures that have associated raw data
- A description of any restrictions on data availability

The data that support the findings of this study are available from the corresponding author upon reasonable request.

# Field-specific reporting

Please select the one below that is the best fit for your research. If you are not sure, read the appropriate sections before making your selection.

☒ Life sciences ☐ Behavioural & social sciences ☐ Ecological, evolutionary & environmental sciences

For a reference copy of the document with all sections, see nature.com/documents/nr-reporting-summary-flat.pdf

# Life sciences study design

All studies must disclose on these points even when the disclosure is negative.

| | |
|---|---|
| Sample size | MEA recordings were typically made in 3-8 retinas, each providing ~20-50 units.<br>Behavior experiments were done on 4-17 animals from each group (usually 5-10). |
| Data exclusions | For all behavior analysis (light dark, pattern association/avoidance, and open field) animals are not excluded from data if they complete task. In all of the experiments presented, only 1 animal from (m-opsin) was removed from pattern association parallel bars for never moving from entry corner of the behavior apparatus. |
| Replication | MEA recordings in Figs. 1 h-i, 2a-e and 4a-c were originally made in response to light flashes of 1s duration to create a complete data set. The experiments were repeated using light flashes of 0.1s duration. The results using the shorter light flash were virtually identical to the original ones with the longer flash, and these shorter ones are reported in the paper. |
| Randomization | Prior to treatment, animal cohorts were divided randomly to age-matched control groups and treated groups. Animals were run in small batches, with groups of control and test animals run in alternation. Groups within a cohort were run in different weeks to ensure that environmental changes within the animal facility testing room did not affect outcomes. Additionally, in some experimental rounds the control group was run first followed by the test group and in others the reverse sequence was used. All experiments were done using computer data analysis. Behavior was measured by IR sensors or video by automated routines that track animal position. |
| Blinding | The experimenter was not blind to the identity of the animals. |

# Reporting for specific materials, systems and methods

We require information from authors about some types of materials, experimental systems and methods used in many studies. Here, indicate whether each material, system or method listed is relevant to your study. If you are not sure if a list item applies to your research, read the appropriate section before selecting a response.

## Materials & experimental systems

| n/a | Involved in the study | |
|---|---|---|
| ☒ | ☐ | Antibodies |
| ☒ | ☐ | Eukaryotic cell lines |
| ☒ | ☐ | Palaeontology |
| ☐ | ☒ | Animals and other organisms |
| ☒ | ☐ | Human research participants |
| ☒ | ☐ | Clinical data |

## Methods

| n/a | Involved in the study | |
|---|---|---|
| ☒ | ☐ | ChIP-seq |
| ☒ | ☐ | Flow cytometry |
| ☒ | ☐ | MRI-based neuroimaging |

## Animals and other organisms

Policy information about studies involving animals; ARRIVE guidelines recommended for reporting animal research

| | |
|---|---|
| Laboratory animals | make and female mice: wildtype and rd1 |
| Wild animals | none |
| Field-collected samples | none |
| Ethics oversight | UC Berkeley OLAC and ACUC |

Note that full information on the approval of the study protocol must also be provided in the manuscript.
